# Computational Studies on Acetylcholinesterases

**DOI:** 10.3390/molecules22081324

**Published:** 2017-08-10

**Authors:** Yechun Xu, Shanmei Cheng, Joel L. Sussman, Israel Silman, Hualiang Jiang

**Affiliations:** 1 CAS Key Laboratory of Receptor Research, Drug Discovery and Design Center, Shanghai Institute of Materia Medica, Chinese Academy of Sciences (CAS), Shanghai 201203, China; smcheng@simm.ac.cn (S.C.); hljiang@simm.ac.cn (H.J.); 2 Israel Structural Proteomics Center, Weizmann Institute of Science, Rehovot 76100, Israel; joel.sussman@weizmann.ac.il; 3 Department of Structural Biology, Weizmann Institute of Science, Rehovot 76100, Israel; 4 Department of Neurobiology, Weizmann Institute of Science, Rehovot 76100, Israel; Israel.Silman@weizmann.ac.il

**Keywords:** acetylcholinesterase, computational modeling and simulation, active-site gorge, ligand trafficking, oligomer, catalytic reaction mechanism

## Abstract

Functions of biomolecules, in particular enzymes, are usually modulated by structural fluctuations. This is especially the case in a gated diffusion-controlled reaction catalyzed by an enzyme such as acetylcholinesterase. The catalytic triad of acetylcholinesterase is located at the bottom of a long and narrow gorge, but it catalyzes the extremely rapid hydrolysis of the neurotransmitter, acetylcholine, with a reaction rate close to the diffusion-controlled limit. Computational modeling and simulation have produced considerable advances in exploring the dynamical and conformational properties of biomolecules, not only aiding in interpreting the experimental data, but also providing insights into the internal motions of the biomolecule at the atomic level. Given the remarkably high catalytic efficiency and the importance of acetylcholinesterase in drug development, great efforts have been made to understand the dynamics associated with its functions by use of various computational methods. Here, we present a comprehensive overview of recent computational studies on acetylcholinesterase, expanding our views of the enzyme from a microstate of a single structure to conformational ensembles, strengthening our understanding of the integration of structure, dynamics and function associated with the enzyme, and promoting the structure-based and/or mechanism-based design of new inhibitors for it.

## 1. Introduction

Alzheimer’s disease (AD) is a neurodegenerative disorder associated with impairments in cholinergic transmission that leads to memory loss [1]. The cholinergic hypothesis argues that enhancing levels of the neurotransmitter acetylcholine (ACh), or inhibiting its degradation, should improve cognitive function. Acetylcholinesterase (AChE) is the enzyme responsible for degradation of ACh in the synaptic cleft at the neuromuscular junction and at cholinergic synapses in the central nervous system (CNS). As shown in Scheme 1, AChE is first acetylated by ACh, with concomitant release of choline. Then, a water molecule regenerates the free enzyme, with concomitant release of acetic acid [2,3]. Due to its prominent role in the nervous system, AChE is the target of a variety of potent natural and synthetic compounds, ranging from synthetic organophosphorus (OP) nerve agents and insecticides to the first generation of anti-Alzheimer drugs [3,4,5]. The latter were developed since, in terms of the cholinergic hypothesis, they have the potential to ameliorate the cognitive deficits associated with the disease.

**Figure 1:**
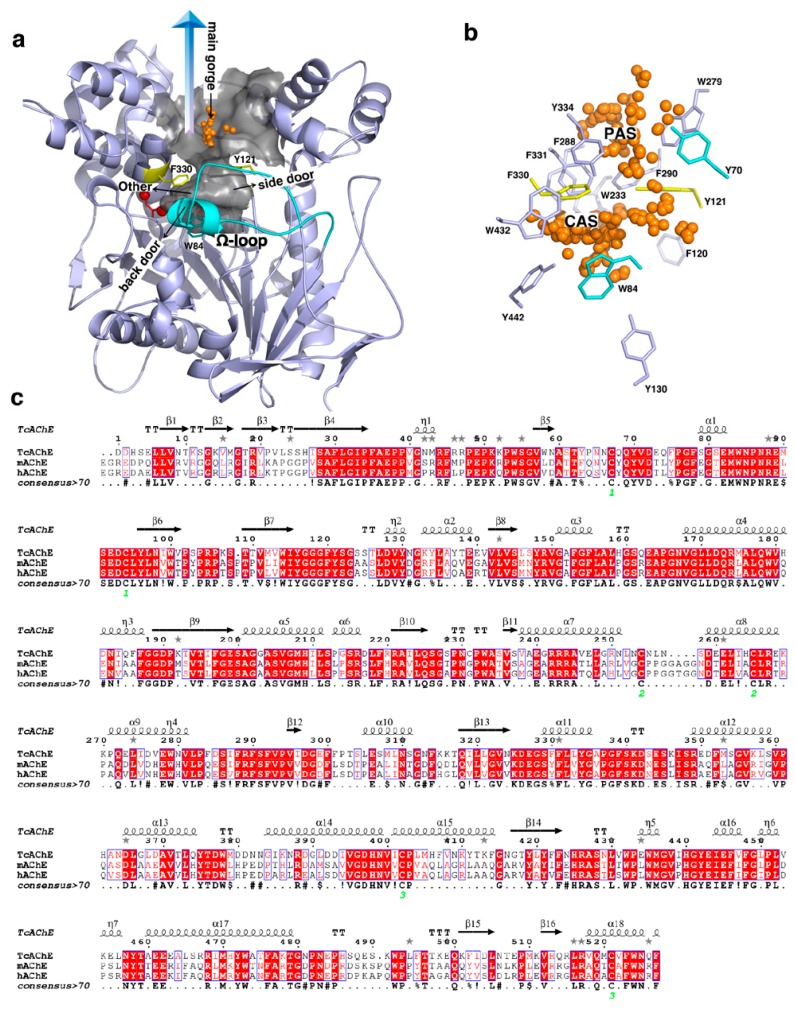
Structure and sequence of AChE. (**a**) A crystal structure of *Tc*AChE (pdb code 2c5g). Stereoview of the active-site gorge and three putative exit pathways for ligand trafficking. The inter-molecular surface of the gorge was generated by PyMol [14]. The probes (orange balls) for detecting the gorge were generated by Fpocket [15]. F330 and Y121 at the bottleneck, and W84 in the Ω-loop near the “back door”, are shown as sticks. The catalytic triad (H440-E327-S200) is shown as spheres with side-chain as sticks. The light-blue arrow indicates the direction of the dipole; (**b**) 14 aromatic residues inside the active-site gorge of *Tc*AChE and two ligand-binding sites: PAS and CAS. The orange balls are the probes for detecting the active-site gorge, generated by Fpocket; (**c**) Multiple sequence alignments of the catalytic domain of *Tc*AChE, *m*AChE and *h*AChE generated using Clustalx [16] and the ENDscript server [17].

The first crystal structure of AChE, that of the enzyme purified from *Torpedo californica* (*Tc*AChE), was reported in 1991 [6]. It shows that a long and narrow active-site gorge, about 5 Å wide and 20 Å deep, extends from the surface of the enzyme down to the catalytic site (Figure 1a). The gorge has two distinct ligand binding sites: the “anionic” sub-site of the catalytic site (CAS), and the peripheral “anionic” site (PAS) (Figure 1b). The CAS, located near the bottom of the gorge, contains a catalytic triad (H440-E327-S200) and a key aromatic residue, W84. The PAS is close to the entrance of the active-site gorge, and W279 is an important component of this anionic site. In the middle of the gorge, a bottleneck formed mainly by the side-chains of F330 and Y121 is so narrow that it only admits passage of a water molecule. Because of its narrow cross-section, substrates and inhibitors with a much larger cross-section than the bottleneck would have no access to the catalytic site if the enzyme were rigid. This intrinsic property is shared by AChEs from other species such as mouse [7] and human [8] AChEs, due to their high sequence similarity to *Tc*AChE, including especially high conservation of residues inside the gorge (Figure 1c). The active-site gorge has two additional remarkable features [6,9,10]. One is the high aromatic content of the walls and base of the gorge (Figure 1b). The other feature is a strong electrostatic dipole, aligned precisely along the gorge axis, produced by the overall distribution of charge residues in the whole catalytic domain, attracting positively charged ligands to penetrate into the active site. It has been suggested that these two features may work together [11]. The dipole provides a driving force while the multiple aromatic groups serve as a series of low-affinity binding sites for the positively charged ligands. In this way, the tunnel leading to the active site acts as a “vacuum cleaner” ensuring a fast supply of the cationic substrate to the active site.

Although the large number of crystal structures of AChE determined for both the apo enzyme and for complexes and conjugates have provided considerable insight into its functions, these insights cannot completely answer such questions as how substrate and other ligands enter and leave the active site, which, in the crystal structures, appears to be accessible only via the long gorge, with its narrow bottleneck referred to above. As a consequence, structural fluctuations are required for the enzyme to operate. Computational studies can supplement the crystallographic data, and complement the information provided by kinetic studies. Molecular dynamics (MD) simulations, derived on the basis of the classic Newton laws, are widely used to describe the dynamic properties relevant to protein function and regulation that cannot be accessed by the “static” crystal structures [12]. With the enormous growth both in the number of protein crystal structures and in computational power, increasingly reliable MD simulations have been performed to explore the relationships between structure, dynamics, and function of proteins at the atomic level [13]. Early simulations on AChE, with short sampling times, focused on the dynamics of the gorge, including changes in its shape and size, side-chain movement of key residues, and the role of water molecules inside the gorge. Later, the binding of the substrate and other ligands into the gorge were investigated with MD and/or Brownian dynamics (BD) simulations. However, the timescale of a physiological event, such as ligand trafficking inside the enzyme, is often too large for classical MD simulations to achieve. Accordingly, in addition to the conventional MD simulations, several modified simulation methodologies or strategies have been employed, including multiple parallel nanosecond MD simulations, multiple copy sampling, umbrella sampling, steered MD simulation, targeted MD simulation, and metadynamics simulation. These allow more efficient exploration of AChE's conformational space, so as to study ligand binding to and/or release from AChE, as well as the dynamic features of oligomeric AChE. In addition, quantum mechanics combined with molecular mechanics (QM/MM) have been used to precisely elucidate both the reaction mechanism of ACh hydrolysis and covalent modification by certain OP compounds. 

In the following, we briefly recapitulate the main findings obtained in recent computational research on AChE, demonstrating the power and advantages of a variety of computational methods in interpreting the experimental observations, and in providing insight into the internal motions of the enzyme at the atomic level. 

## 2. Results

### 2.1. Features of the Active-Site Gorge 

#### 2.1.1. Gating of the Active-Site Gorge

The active-site gorge is the most noteworthy feature of AChE. A series of MD simulations have observed changes in the gorge’s dimensions, in particular in the size of the bottleneck, mainly contributed by F330 and Y121 in the middle of the gorge, which varies during the simulations. On the basis of an early 500-ps simulation of the *Tc*AChE-tacrine complex it was seen that the gorge fluctuates rapidly between closed and open states up to ~0.52 nm in diameter, large enough to permit the passage of the natural substrate [18]. However, the gorge was open for less than 5% of the total simulation time. In another simulation study, using a 2.4 Å probe to model ACh, only ~2.4% of the total 750 ps was detected as being in the open state [19]. However, the quantitative analysis reveals that the rate of substrate binding is slowed only by a factor of 2. The explanation for this result may be that the substrate makes repeated attempts to pass through the gate due to Brownian motion, while the gate is rapidly switching between the open and closed states. Accordingly, when one of these repeated attempts coincides with an open state, the substrate will succeed in entering. A more accurate description of the dynamics of the bottleneck region of the active-site gorge was carried out with a 10-ns MD simulation of the monomeric *m*AChE [20]. The gorge size was quantified with a single variable, the gorge radius, ρ(t), for snapshots recorded every picosecond from the MD trajectory. The gorge radius for each snapshot was defined as the radius of the largest spherical ligand that can pass through the bottleneck and reach the active-site. The resulting average gorge radius was 1.5 Å. ρ(t) was restricted within a range of 0.8 to 2.4 Å, with a standard deviation of 0.26 Å [20]. Very recently, microsecond MD simulations of the *Tc*AChE-E2020 complex and/or of the *Tc*AChE dimer have shown that the occupancy of the open state of the gorge increased from 0.2% in the monomer to 13.8% in the complex, to 20.6% and 17.5% in the two chains of the apo dimer, and to 50.5% and 40.6% in the two chains of the E2020-complexed dimer [21]. The effects of ligand binding and dimerization on gorge motions seem to be cooperative, since the largest occupancy of the open state was seen in the complexed dimer.

#### 2.1.2. The Role of the Electrostatic Potential in Binding of Cationic Ligands

AChE is an extremely effective catalyst, acting on ACh at rates approaching those associated with diffusion-controlled reactions, meaning that the encounter of the substrate with the enzyme is rate limiting. The crystal structure of *Tc*AChE revealed an asymmetric charge distribution that results in an electrostatic field that may accelerate the encounter of a cationic ligand with the entrance to the active site. Accordingly, a series of Brownian dynamics (BD) simulations were carried out by Antosiewicz and colleagues to test the hypothesis of electrostatic steering [22,23,24]. The BD simulation, a diffusional analogue of MD, was processed with the numerical integration of the Langevin equation [25,26]. It is widely used to study enzyme-substrate or enzyme-inhibitor association, the effect of site-specific mutations, and perturbations of pH, viscosity and ionic strength on bimolecular diffusional association rate constants. In comparison with MD or QM/MM calculations, it is less computationally exhaustive. 

A BD simulation of the diffusion of a dumbbell-shaped ligand, whose diffusion properties and charge distribution approximate those of ACh, in the electrostatic field of *Tc*AChE, found that the field indeed guides the substrate to the mouth of the active-site gorge [22]. However, the discrepancy between the calculated and experimentally measured rates suggested that structural fluctuations in the enzyme or events subsequent to the encounter may contribute to rate limitation. Another BD simulation on a mutant *Tc*AChE in which the surface acidic residues homologous with those mutated in the *h*AChE [27] were artificially neutralized, again found that the electrostatic field of the enzyme increases the rate constants by about an order of magnitude in both the wild-type and the mutants [23]. Soon afterwards, Antosiewicz et al. presented a more comprehensive study which revealed that the electrostatic steering is limited to relatively short distances from the surface of the enzyme [24]. In addition, not only the total charge but also the dipole moment is responsible for the electrostatic steering of a cationic ligand to the active site, and mutations that only affect one of these two factors may not change the calculated encounter rate constants. Remarkably, based on multiple BD trajectories, Zhou et al. predicted that the electrostatic potential within the active-site gorge should increase the *Tc*AChE-ACh binding rate by 240-fold [28]. 

**Figure 2:**
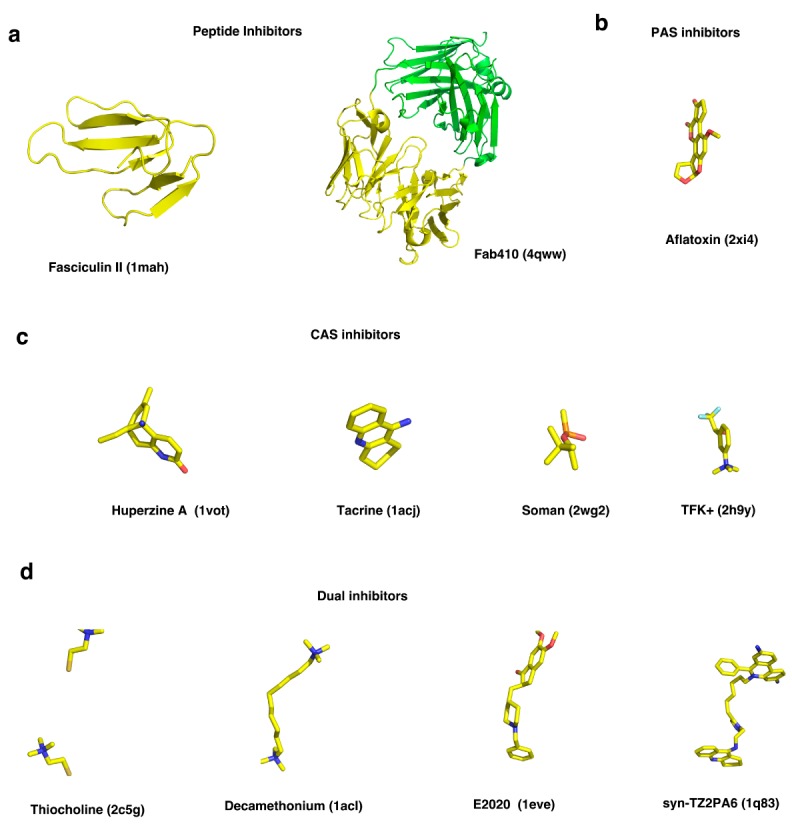
Structures of AChE inhibitors. (**a**) peptide inhibitors; (**b**–**d**) inhibitors binding at the PAS (**b**), the CAS (**c**) and both sites (**d**). The corresponding pdb codes of the crystal structure of the AChE complexes of the inhibitors are shown in brackets.

To explore the role of surface and active-site charged residues in electrostatic attraction of ligands to the active center gorge of *m*AChE, as well as in determination of the reactive orientation of a ligand, the kinetics of binding of a small cationic ligand (*m*-trimethylammoniotrifluoroacetophenone, TFK^+^, Figure 2) and a polypeptide inhibitor (fasciculin II, FAS2) with the CAS and the PAS of *m*AChE, respectively, were investigated by Radić and coworkers based on enzymic activity assays complemented by BD simulations [29]. It was demonstrated that reduction in ionic strength strongly enhances the rate of association of FAS2 with *m*AChE, and that the effect can be distinctly reduced by progressive neutralization of surface and active-site gorge anionic residues. By contrast, neutralization of multiple anionic residues (E84, E91, D280, D283, E292, and D372) on the surface only has a modest effect on the rate of cationic TFK^+^ binding to the active-site serine, whereas anionic residues within the gorge such as D74, near the rim, and E202 and E450, near the base, are likely to be critical for guiding the cationic ligand into the gorge and for achieving the reactive orientation of the ligand [29,30]. D74 was shown to play a particularly important role in restricting motions of the ligand inside the active-site gorge [30]. Noteworthy, the dissociation of both TFK^+^ and FAS2 is insensitive to ionic strength, suggesting that the electrostatic potential does not have as significant an influence on the ligand’s dissociation as it does on its association. Another BD simulation on the association of FAS2 with *m*AChE, carried out by Elcock and colleagues, revealed that electrostatic interactions do promote fast association of the polypeptide inhibitor with the enzyme, but that electrostatic desolvation effects, resulting from exclusion of the high dielectric solvent that accompanies the approach of the two low dielectric proteins, appear to be important for reproduction of experimentally measured association rate constants in addition to the ionic strength effects [31]. Incorporation of an approximate description of the electrostatic desolvation effects into a second set of BD simulations made the calculated association rate constants much closer in magnitude to the experimental ones.

#### 2.1.3. Flexibility of the Aromatic Residues inside the Gorge

A remarkable feature of AChE is the high aromatic content of the deep and narrow active-site gorge. For example, the *Tc*AChE gorge is lined by 14 conserved aromatic amino acids, F120, F288, F290, F330, F331, W84, W233, W279, W432, Y70, Y121, Y130, Y334, and Y442, the aromatic rings of which contribute ~60% of the total surface area of the gorge [6]. These residues are highly conserved, and most of them can be assigned functional roles. W84 and F330 are involved in the so-called “anionic” sub-site of the CAS, making π-cation interactions with the quaternary group of the substrate; W279 and Y70 contribute to the PAS. According to the degree of flexibility of the aromatic side-chains, the 14 aromatic residues can be divided into three groups [32]. Residues F120, F331, F288, F290, W233, W432, Y70, and Y121 (group I) fall into a category for which both the crystallographic data and MD simulation trajectories indicate very low flexibility. In contrast, for F330 and W279 (group II), the MD trajectories and the crystallographic data indicate high flexibility of their side-chains. The third category (group III) includes W84, Y130, Y442, and Y334, whose side-chains appear being quite flexible in the 20-ns MD simulations, even though no substantial flexibility is evident in the crystal structures. 

Butyrylcholinesterase (BChE) is a closely related enzyme to AChE, with high sequence homology to *Tc*AChE [33]. Six of the fourteen conserved aromatic residues in the gorge of *Tc*AChE are substituted by smaller aliphatic or even polar residues in BChE, accounting for most of the differences in catalytic properties of the two enzymes. In BChE, there is no cluster of aromatic residues at the PAS, and no aromatic bottleneck exists midway down the gorge to limit ligand trafficking, while the acyl-binding pocket is larger than that in AChE, allowing accommodation of a substrate with a longer acyl chain. MD simulations of monomers and tetramers of *h*AChE and *h*BChE in water-solvated systems found that the different gating mechanisms of the active-site gorges of the two homologous enzymes are responsible for their distinct substrate preferences [34]. It was also suggested that the tetramer of both enzymes might have two dysfunctional active sites due to their restricted accessibility to substrates [34]. In an early study on conversion of AChE to BChE, the site-directed mutagenesis of two residues, F288L and F290V, produced a double mutant that hydrolyzed butyrylthiocholine almost as well as acetylthiocholine, while the W279A mutant displayed significant reduced inhibition by the PAS-specific ligand propidium but not by the CAS-specific inhibitor edrophonium [33]. Moreover, gradual replacement of the 6 aromatic residues in *h*AChE by the corresponding residues in *h*BChE resulted in a hexa-mutant *h*AChE (Y72N/Y124Q/W286A/F295L/F297V/Y337A) that displayed reduction in affinities for tacrine, decamethonium, edrophonium, huperzine A (HupA), and BW284C51, compared with those of the wide-type *h*AChE. Although the hexa-mutant *h*AChE does not mimic the reactivity of *h*BChE toward substrates and other covalent ligands, it displays a reactivity phenotype closely resembling that of *h*BChE for most of these prototypic noncovalent inhibitors [35].

#### 2.1.4. Flexibility of the Ω-Loop

The Ω-loop is an important loop between two cysteines (C67–C94 of *Tc*AChE and C69–C96 of *m*AChE or *h*AChE) (Figure 1c) that participates in the active-site gorge, the back door and the side door of the enzyme (Figure 1a). Both experimental and theoretical studies revealed that this loop is flexible and contributes to transient gorge enlargements that could facilitate ligand binding and release. An early spectrofluorometric study suggested that association of the reversible inhibitors and phosphorylation or carbamoylation of the active-site serine both induced large conformational changes of the loop in *m*AChE [36]. A 15-ns MD simulation, together with site-directed labeling in conjunction with time-resolved fluorescence anisotropy, revealed that the Ω-loop of *m*AChE is capable of undergoing residue-specific and ligand-dependent changes in backbone motions since binding of both FAS2 and HupA, with different sizes and binding modes, affected the flexibilities of different residues in the loop [37]. However, the residue dynamics and the backbone movement of the loop are not tightly coupled, indicating that transient gorge enlargements result from random or near random (non-concerted) fluctuations that periodically widen the gorge in a time frame of nanoseconds or sub-nanoseconds [37]. In addition, MD simulations of *m*AChE in complex with FAS2 showed that the Ω-loop undergoes a flexible rearrangement during which some of its secondary structure rapidly changes from a coil to a 3_10_ or α helical segment [38]. This arrangement at the tip of the long Ω-loop generates a hydrophobic patch (L76-F80 in *m*AChE) by exposing its aromatic side-chains to the solvent, which might provide a structural basis for interactions between the β-amyloid peptide and AChE [39]. In our studies on the flexibility of aromatic residues in the active-site gorge of *Tc*AChE, it was also found that the Ω-loop is mobile in the MD simulation while it is quite fixed in the crystal structures of AChE, mostly due to restraints imposed by crystal packing [32]. The backbone movements of the Ω-loop not only cause W84 to adopt different side-chain orientations but may also affect the side-chain conformations of the other four aromatic residues with which it is interacting, namely Y130, Y334, W432, and Y442. In a second simulation with the application of force restraints to the main-chain atoms of the Ω-loop, the side-chain flexibility of W84, as well as of the other four residues, is substantially reduced compared to the case in which the main-chain of the loop is not restrained.

#### 2.1.5. Properties of Water Molecules inside and outside the Active-Site Gorge

The functions of water molecules in proteins have attracted a lot of attention [40]. AChE and its complexes with inhibitors contain a large number of buried water molecules, twice the number per residue typical for globular proteins. Compared to the bulk water, these gorge water molecules display a number of properties that may aid ligands entry and exit, including fluctuations in the population of water molecules inside the gorge, moderating disorder and mobility of water in the middle and entrance to the gorge, reducing the water-mediated hydrogen-bonding ability, and transient existence of cavities inside the gorge [41]. Koellner et al. analyzed structural aspects of the buried water molecules in the catalytic domain of *Tc*AChE and of the water molecules within its active-site gorge by comparing five crystal structures, suggesting that large arrays of buried water molecules acting as a lubricant that permit or even facilitate large-scale fluctuations of the loops forming the active-site gorge, and water molecules in the active-site gorge are more energetically activated compared to bulk ones [42]. Our previous steered MD simulations showed that water molecules in the active-site gorge facilitate HupA’s entrance into or exit from the active site by comparing the pulling force, which decreased from ~1500 pN in a vacuum to ~800 pN in aqueous solution [43].

To distinguish the hydration shell of water from the bulk water outside the gorge, properties of water molecules less than 3.6 Å from the protein surface were studied by MD simulations [44]. The water dynamics network of the hydration shell is not affected by size, function and secondary structures, as found by comparison of four globular proteins, AChE, subtilisin Carlsberg, lysozyme, and ubiquitin [45]. The similarity can be ascribed to the similar local surface topology and surface chemical composition of these four proteins. However, the dynamics of the water network was influenced by binding of a non-covalent inhibitor, HupA, or a covalent inhibitor, soman, to the active site of *h*AChE [46]. Soman forms a covalent bond with the catalytic serine of *h*AChE. This conjugate undergoes a spontaneous dealkylation called “aging” that leads to the establishment of a stabilizing salt bridge between the aged phosphonate moiety and the protonated active-site histidine [47]. Such a salt bridge makes the enzyme more stabilized or stiff, and is accompanied by release of free water molecules, so that it indirectly affects the dynamics of the hydration shell [46].

### 2.2. Ligand Trafficking Pathways: Back Door and Side Door

Ever since the first AChE structure was determined by the X-ray crystallography [6], it was difficult to understand how the long and narrow active-site gorge can be reconciled with the enzyme’s remarkably high catalytic rate. The mechanisms of the rapid binding of substrate and inhibitors became a key area of interest, as did that of product release. In addition to the active-site gorge, alternative routes to facilitate traffic of ligands or solvents into and out of the gorge have been suggested. Several MD simulation studies have been performed to investigate the release pathways for small molecules such as acetic acid, choline and water molecules. Initially, a short MD simulation (~119 ps) of native *Tc*AChE in aqueous solution revealed transient opening of a short channel near W84, the so-called back door, connecting the CAS to the bulk [48]. It was indicated that such a back door for the escape of water molecules or reaction products might be fostered by a local conformational change near the active-site cavity including residues W84, V129, G441, and Y442 (Figure 1a). Later, 1-ns MD simulations of *m*AChE without or with the inhibitor HupA bound at the CAS also visualized the opening of the back door [49]. Several years ago, we performed multiple conventional MD simulations to investigate the clearance of thiocholine (TCh), a mimic of the reaction product choline, from the active-site of *Tc*AChE, thus revealing that the back-door pathway is most frequently used for trafficking of TCh [50]. It was found that the shutter-like motion of the aromatic side-chain of W84 is crucial for TCh to exit the CAS via the back door. A side-chain rotation of Y442, breaking the hydrogen bond linking it to W84, was found in the crystal structure of *Tc*AChE in complex with the PAS-binding inhibitor aflatoxin (Figure 2), resulting in a 3.4-Å wide back door (PDB code 2XI4) [51]. A MD simulation starting with this structure shows that Y442 moves further away from W84, permitting the back door to open more widely and frequently [51]. Additional experimental evidence for an open back door was found for *Drosophila melanogaster* AChE (*Dm*AChE), in which mutation of W83 to Ala or Glu widens the back-door channel [52]. In addition, the residual catalytic activity observed in complexes of AChE with FAS2 or with monoclonal antibodies, which almost completely block access to the active-site by tightly binding to the PAS, lent support to the back-door hypothesis [7,53,54,55,56]. Simulations of the dynamic behavior of *m*AChE-FAS2 complexes or of the binding of FAS2 to *m*AChE indicate that the back-door passage is open most of the time [57,58].

Besides the back door, other ligand trafficking pathways have been observed in numerous simulation studies. Utilizing MD simulations, the McCammon group discovered that a side door located ~15 Å from catalytic S200, is approximately perpendicular to the gorge entrance, and is composed of residues from the Ω-loop (residues 67 to 94) of *Tc*AChE (Figure 1a) [18,38]. A 500-ps MD simulation of the *Tc*AChE dimer liganded with the CAS inhibitor tacrine in solution was performed [18], disclosing that, in addition to the active-site gorge, alternative entries to the active site through the side wall exist transiently which permit exchange of water molecules [18]. The polypeptide toxin FAS2 binds at the entrance of the active-site gorge (Figure 2). A 15-ns MD simulation of *m*AChE in complex with FAS2 revealed that the structural fluctuations of this complex substantially increase compared to the native enzyme, and the flexibility of the long Ω-loop is particularly enhanced, resulting in additional routes of access to the active-site, such as the so-called side door [38].

A recent MD simulation study of soman-modified *h*AChE revealed that both the back- and side-door pathways are viable for soman access [59]. Moreover, the motion correlation between the active-site gorge and the back door is disrupted after the enzyme has been modified by soman, suggesting that substrate and product can traffic via different pathways. This is consistent with the conclusion reached from the MD simulations for the *Tc*AChE-tacrine complex, namely that the bound tacrine interferes with entrance into the active-site gorge, so that water molecules appear to access the active-site through alternative pathways [18].

Other doors, such as one through the W279-F290 loop of *Tc*AChE, on the opposite side of the active site from the Ω-loop, have also been proposed (Figure 1a) [60]. The flexibility of this loop may permit the binding of bulky inhibitors such as galanthamine. It is also observed in the exit of small molecules such as ammonium and methane from *Tc*AChE in multiple copy simulations [61]. The MD simulation with the soman conjugate (Figure 2) exhibits the flexibility of the W279-F290 loop, and provides support for the existence of the corresponding door [59].

### 2.3. Ligand Trafficking within AChE

#### 2.3.1. ACh, Choline and their Analogues

In order to study the ligand binding specificity of AChE prior to the availability of relevant crystal structures, docking of ACh, ACh analogues and choline to multiple snapshots extracted from the apo-*m*AChE and ACh-*m*AChE MD trajectories were carried out [62,63]. It was revealed that the bound ACh in the active site not only stabilizes the catalytic triad, but also tightens the interaction between the esteratic sub-site, containing the catalytic machinery, and the “anionic” sub-site, so as to achieve better binding of the substrate. Both the size and the positive charge of the quaternary tetramethylammonium (TMA) group are important for its binding to the enzyme. E202 at the anionic sub-site was found to stabilize binding of ACh via electrostatic interactions. However, the size/shape complementarity between the anionic sub-site and the TMA group makes an even larger contribution than the electrostatics, as any perturbation (including residue mutagenesis) to the anionic sub-site residues, W86, E202 and Y337, results in a significant reduction in binding of the extended ACh in the catalytically productive orientation [63].

To study binding to and dissociation from AChE, the interaction of TMA, a simple ligand that can cross the bottleneck, was first studied by the umbrella sampling and active flux techniques [64]. Umbrella sampling addresses systems with energy landscapes described by sets of collective variables, with each set determining a separate umbrella window [65]. In each window, a restraint bias quadratic or harmonic formed potential is applied to force the collective variables to remain close to the mass center. TMA resembles the bulkiest part of ACh, and they both contain a quaternary ammonium group. The free energy profile calculated based on the simulation showed that the barrier created by the bottleneck is about 4 kcal/mol, which is consistent with the fast reaction rate of the enzyme. Small and fast fluctuations of the aromatic side-chains in the bottleneck region, together with the large-scale collective motions of the enzyme, are coupled with the movement of the ligand. 

Multiple copy sampling, also termed locally enhanced sampling, is an approach based on the classical time-dependent, self-consistent mean-field approximation [66]. By making use of this method, the clearance of seven ligands from *Tc*AChE was studied [61]. It was found that different ligands might travel via different pathways. For small ligands such as ammonium or methane, exit from the gorge was found to occur via several routes. Whereas the polar ligands, methylammonium and acetic acid, left their binding sites solely via the active-site gorge. Bulkier ligands, such as TMA and neopentane, as well as the smaller acetate ion, remained trapped inside the active site. 

More recently, we utilized conventional MD simulations to explore the traffic of TCh within the active-site gorge of *Tc*AChE [50]. TCh is generated enzymatically by hydrolysis of the chromogenic substrate, acetylthiocholine, and it displays kinetic properties virtually identical to those of ACh. Three simulation groups were generated, in which TCh was bound at the CAS, at the PAS, or at both sites. Forty 20-ns trajectories were run for each simulation group. The statistical results from these multiple parallel MD simulations indicated that TCh is released from the PAS via random pathways, while three exit routes appear to be favored for its release from the CAS, namely, along the axis of the active-site gorge, and through putative back and side doors. The back-door pathway is that via which TCh exits most frequently. The side-chain flexibilities of F330 and W84, and interactions of TCh with these two residues are shown to be crucial for TCh to traffic through the active-site gorge and the back door, respectively.

#### 2.3.2. HupA

Steered MD (SMD) simulation, an extended MD simulation method mimicking the principle of atomic force microscopy, has been widely used to explore the binding and dissociation of biomolecules with external mechanical manipulations [67]. Time-dependent external force is usually applied to the ligand to facilitate its binding to or dissociation from the target protein. On the basis of the accelerated movement of the ligand, the enzyme’s flexibility and its response to association or dissociation of the ligand can be investigated. Numerous SMD simulations were carried out with AChE-ligand complexes [43,68,69,70]. Analysis of the interactions between the moving ligand and the enzyme, and study of the relationship between the applied force and the ligand’s position, can yield fresh insights into structure-function relationships of AChE in general, and more specific information concerning the pathways of association and dissociation of the ligand. 

HupA is an alkaloid isolated from the Chinese herb, *Huperzia serrata*, which has been used in Traditional Chinese Medicine (TCM) for many centuries to treat contusions, strains, swellings, and schizophrenia [71]. HupA is a strong AChE inhibitor, and has attracted considerable interest due to its low toxicity, high selectivity and long duration of inhibition [71,72,73]. Thus, upon dilution, the HupA-inhibited AChE complex takes several hours to dissociate, and to recover enzymic activity. This phenomenon is in contrast to the rapid on- and off-rates that characterize most reversible inhibitors of AChE with similar potency. It is thus assumed that HupA has a longer residence time than other anti-AChE drugs that have been used for the treatment of Alzheimer’s disease [43,74]. The entry of HupA into the active-site gorge of *Tc*AChE and its exit have been investigated using SMD simulations [43]. The rupture force for pulling HupA out of the active site is much larger than the force required to push it into the active site, which is in good agreement with the aforementioned experimental evidence that the association of HupA with *Tc*AChE is much quicker than its dissociation [74]. Breakage of direct hydrogen bonds of HupA with residues at the CAS results in requirement for a large pulling force in the dissociation process. The steric hindrance of bottleneck may be the major factor in the requirement for large rupture force for HupA to exit the binding site, but it has little effect on the binding process. Residue D72, located between the PAS and the CAS in *Tc*AChE, is a key residue with which ligands interact. It acts as a clamp both in extracting HupA from the active site and in inserting it. Earlier studies showed that while D72 contributes little to the overall electrostatic effect on cationic ligands, its mutation reduced their dissociation constants [29,75]. When HupA leaves or enters the active-site gorge, it forms multiple water bridges that may serve as lubricants that permit or even facilitate trafficking of HupA and other ligands within the gorge. The fact that D72 is involved in many such interactions explains its role as a clamp. An SMD simulation of HupA leaving the *Tc*AChE gorge in which water molecules were omitted resulted in a dramatically increased pulling force, validating the lubricative role of the water molecules, in particular those within the gorge.

To better understand the ligand-target binding process, a new computational method was developed to generate a rigorous and precise free energy landscape for ligand binding (BFEL) [76]. With such an energy landscape, both the binding affinity and the binding kinetics of a ligand interacting with its target protein can be accurately calculated. A complete free energy landscape of HupA binding to *Tc*AChE was constructed, from which the binding free energy and the activation free energies for association and dissociation are quantitatively estimated. The calculated values of all three terms are in excellent agreement with data derived from surface plasmon resonance measurements. In particular, the higher activation energy for dissociation than for association matches convincingly with the finding mentioned above that HupA binds to AChE much more quickly than it disassociates [43,74]. Qualitatively, the lowest-energy pathway for HupA binding to *Tc*AChE derived from the BFEL calculations has three low-energy wells that correspond to three metastable states (B0–B2), one stable state (B3), which closely resembles the crystal structure of the *Tc*AChE-HupA complex, and two energy peaks related to two transition states (P1 and P2). The highest-energy barrier (about 35.5 kJ/mol) is produced when HupA has to overcome the strong steric hindrance from N85, Y70, D72, Y121, F330, Y334, H440, and W84, close to the second transition state, P2.

#### 2.3.3. E2020

E2020 (((*R*,*S*)-1-benzyl-4-)[(5,6-dimethoxy-1-indanon)-2-yl]methylpiperidine) is a typical bivalent AChE inhibitor (Figure 2). It spans the whole gorge of AChE, binding to both the CAS and the PAS [10,77]. In an SMD study on the interactions of E2020 with *Tc*AChE nanosecond simulations at a variety of pulling velocities yielded dissociation force profiles [68]. At a velocity of 0.005 Å/ps, the important residues associated with E2020 dissociation were identified. Whereas for some other AChE inhibitors studied, such as HupA, the greatest barrier is near the bottleneck, the major obstacle to E2020 leaving the gorge is at the PAS, where E2020 interacts strongly with the aromatic residues and forms direct and water-bridged hydrogen bonds, thus requiring the largest rupture force. It was found that rotation of the bonds between the piperidine ring and both the benzene ring and the dimethoxyindanone moiety facilitate movement of E2020 through the bottleneck by avoiding serious clashes with Y121 and F330. Intriguingly, the aromatic residues lining the active-site gorge wall are the major components making π-cation and/or hydrophobic interactions with E2020. These aromatic residues acting as “senders” and “receivers”, form a conveyer belt for E2020 trafficking through the gorge. Serving as bridges between E2020 and residues within the PAS, water molecules also play a role in disassociating E2020 from *Tc*AChE. Thus, the SMD studies revealed the very different dissociation mechanisms of the two inhibitors, HupA and E2020, at the atomic level, strengthening our understanding of the complex drug–protein interaction, and providing valuable information for drug design.

#### 2.3.4. Fasculin-2

Fasciculin-2 (FAS2), a 61-residue polypeptide with four intrachain disulfide bonds, which belongs to the family of “three-fingered” snake toxins, selectively inhibits mammalian AChEs [7,53]. The positively charged FAS2 binds to the enzyme’s PAS to inhibit catalysis by sterically blocking the active-site gorge entrance and allosterically disrupting the catalytic triad near the bottom of the gorge [7,57]. An early BD simulation study of the binding of FAS2 with *m*AChE suggested that electrostatic interactions play a prominent role in promoting the fast association of the toxin with the enzyme [31]. Subsequently, a 2-ns isobaric-isothermal ensemble MD simulation of FAS2 was performed to examine the dynamic properties which may play a role in its association with AChE [78]. Furthermore, conformations of FAS2 in the presence and absence of AChE as well as the dynamical transitions between these two states were studied by targeted MD simulations, revealing that the intrinsic flexibility of AChE, particularly of the long Ω-loop, facilitates rapid conformational conversion from the free state of FAS2 to its conformation when complexed with AChE. The formation of the AChE-FAS2 complex thus involves an induced-fit mechanism rather than a pre-equilibrium mechanism [58]. The long Ω-loop forms one wall of the active-site gorge and its enhanced fluctuations result in additional routes of access to the active site [38]. When the enzyme is complexed with FAS2, the gorge width distribution is altered so that on the average it becomes narrower. Moreover, there are large increases in the frequency of opening of alternative entrances, namely, the side door and the back door [57].

#### 2.3.5. Interaction of Quaternary Oximes with Conjugates of AChE with Organophosphate Nerve Agents

**Figure 3:**
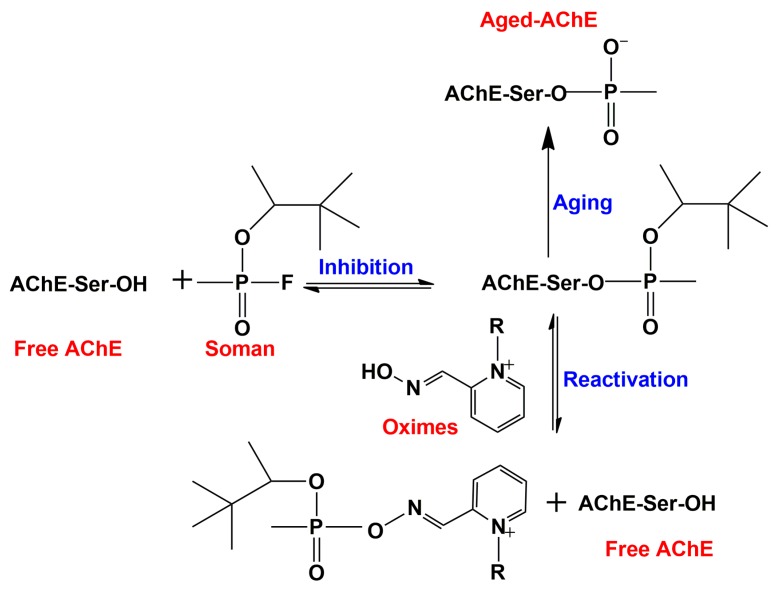
AChE is inhibited by the OP nerve agent, soman, and the inhibited enzyme either is converted to an “aged” OP conjugate, or is reactivated by an oxime.

AChE can be irreversibly inhibited by organophosphorus (OP) nerve agents and insecticides that phosphorylate the active-site serine (Figure 3) [79]. The OP nerve agents, in particular, such as sarin, soman, tabun, and VX, are among the most toxic compounds known, and much effort has been devoted to development of prophylactic and therapeutic treatments of civilians and soldiers who have been exposed to them. A principal element in therapeutic treatment involves the use of mono-quaternary and bis-quaternary oximes that interact with the CAS and displace the OP from the active-site serine (Figure 3) [80]. Such oximes have indeed been successfully used for treatment of OP intoxication. However, “aged” OP-AChE conjugates, in which the bound OP moiety has undergone spontaneous dealkylation (Figure 3), are totally resistant to oxime reactivation, thus presenting a notorious toxicological problem that has yet to be overcome [81]. Efforts to better understand the interaction of quaternary oximes with the OP-AChE conjugates in order to approach this problem have included use of docking and MD approaches. In this context, SMD simulations were performed on the oxime complexes of an “aged” tabun-mAChE conjugate [69] and an “aged” soman-*Tc*AChE conjugate [70]. SMD simulations revealed that two oximes, HI-6 and Ortho-7, with a similar overall structure, bind differently to the tabun-mAChE conjugate [69]. The pyridinium ring of HI-6 shows excellent complementary with the PAS, whereas one of two pyridinium rings of Ortho-7 has excellent binding compatibility with the CAS. Based on these computational data it is predicted that Ortho-7 should be a more efficient reactivator than HI-6. In addition, the dissociation of an oxime-based sulfonium compound from the enzyme was also investigated by SMD simulations, indicating that this newly designed oxime is a potential candidate to reactivate the irreversibly inhibited OP-AChE conjugate [70].

To understand the factors influencing the interactions of fluorinated drugs with their targets, the dissociation of obidoxime (OBI) and of fluorinated obidoxime (FOBI) from the active-site gorge of AChE was studied by use of well-tempered metadynamics simulations [82]. Metadynamics reconstruct the free energy by adding a history-dependent bias potential to the complex Hamiltonians [83,84]. The potential is composed of a sum of Gaussians deposited along the system trajectory in a few selected collective variables. This method greatly enhances rare events in the system studied, and forces the system to escape the energy minima efficiently. Although the dissociation of both oximes is strongly affected by cation-π interactions, hydrogen bonds and water bridges, it is found that distinct dissociation pathways exist for FOBI and OBI. FOBI is more firmly retained in the gorge than OBI, implying that it may have higher reactivation efficiency for the inhibited enzyme. 

#### 2.3.6. *syn*-TZ2PA6

MD simulations were used to elucidate the induced-fit effect of binding *syn*-TZ2PA6 (Figure 2d), an ultra-high-affinity inhibitor with a femtomolar binding affinity, to apo *m*AChE [85]. The initial complex structure used for MD simulation was generated by docking *syn*-TZ2PA6 in its crystallographic binding mode into a large ensemble of MD snapshots of apo *m*AChE. During the first 2 ns, the enzyme undergoes induced-fit conformational changes to yield a structure that is very similar to the crystal structure of *m*AChE-*syn*-TZ2PA6 determined by Bourne et al. [86]. In the process, the side-chain of W286 flipped out of the hydrophobic core and became highly exposed to solvent, and the imidazole ring of H287 assumed an orientation almost orthogonal to its position in the apo enzyme, creating a stable π stacking interaction with the side-chain of W286. Other major conformational changes in active-site residues include side-chain conformational changes of Y133, Y337 and F338, which deviated from their positions in apo *m*AChE to better accommodate the inhibitor in the active-site gorge of the complex. 

### 2.4. Dynamics of Oligomeric AChE

Since the functional forms of AChE are usually oligomers, it is important to consider the oligomeric state of the enzyme when performing MD simulations or when calculating and interpreting catalytic rates. For example, the principal form of *Tc*AChE in the electric organ tissue of *Torpedo californica* is a dimer [87]. A simulation of the *Tc*AChE dimer complexed with tacrine in solution was performed by Wlodek et al. [18]. Within the 100–500 ps time range, a slight approach of two monomers and a small torsional motion of the two subunits relative to each other was detected. Subsequently, on the basis of a 750-ps MD trajectory performed on the *Tc*AChE dimer, the electrostatic potential around it was calculated by solution of the linearized Poisson–Boltzmann (LPB) equation, using the UHBD program [88]. It showed that in both monomers the potential is relatively strong near the gorge mouth, and exerts a rather steady attractive force that steers ACh towards the active site.

Recently, we performed microsecond MD simulations on both the monomer and dimer of *Tc*AChE, as well as on the complex of *Tc*AChE with E2020. Both dimerization and complexation with E2020 increase the gorge radius as well as the opening time of the bottleneck, but also change the dynamic patterns of the residues and subdomains surrounding the active-site gorge. The bound E2020 prevents the active-site gorge from shrinking, and maintains it in a relatively large and stable state. In four simulations performed on apo-*Tc*AChE, E2020-*Tc*AChE, (apo-*Tc*AChE)_2_, and (E2020-*Tc*AChE)_2_., the largest minimal gorge radius was seen in the last one, *viz.*, the E2020 complex of the dimer. The significant influence of dimerization on motions of the active-site gorge is intriguing since the dimerization interface is far away from the gorge. It was shown that the dynamic residues at the C-terminus portion of the enzyme contribute more to the gorge radius variations than the N-terminal residues. The C-terminal residues either directly participate in dimerization or are near the monomer-monomer interaction interface. For example, subdomains S3 and S4, both from the C-terminal portion, are located in and close to the dimer interface, respectively. Allosteric modulation of one subunit by the other could be achieved through subdomains S3 (with or without S4) and the Ω-loop. Accordingly, it is proposed that the effect of the dimer interface on the gorge motions may be achieved by modifying the dynamic pattern of S3 first, and subsequently affecting the dynamics of S4 and/or the Ω-loop, whose movements are both highly correlated with those of S3.

The catalytic subunit tetramer is the most important functional form of *h*AChE, and also in other vertebrate species [89]. To investigate the large-scale inter-subunit dynamic assembly of monomers, all-atom MD simulations combined with Cα-based coarse-grained BD simulations were performed on a full-length, tetrameric model of *h*AChE (*h*AChEt), which was built based on the proline-rich attachment domain interacting with the C-terminal amphipathic t-peptide and a compact tetrameric structure of the catalytic domain [90]. While normal mode analysis suggested that *h*AChEt could exist in several conformations with subunits fluctuating relative to one another, the simulations showed that the tetramer indeed constitutes a dynamic assembly of monomers. The in-plane translation and out-of-plane rotational motions of the four catalytic domains are correlated with dynamic occlusions of the PAS which gate ligand-protein association, and allow the active site to be accessible for more than 80% of the time on average. This was consistent with experiments showing that binding of a ligand to the PAS is only somewhat hindered by the association of the subunits. Therefore, such a combination of methods involving classic atomistic and coarse-grained simulations, as well as normal mode analyses, establishes a link between domain motion and ligand accessibility.

Since the hydrolysis of ACh by *h*AChEt is a typical example of a diffusion-controlled biological reaction catalyzed by an oligomeric enzyme, equilibrium and non-equilibrium electro-diffusion models were further utilized to study the correlation between reaction rate coefficients and enzyme dynamics using an ensemble of structures of *h*AChEt generated by MD combined with BD simulations [91]. With the static initial model, the average reaction rate per active site is approximately 22–30% slower in the tetramer than in the monomer. Such a major effect of tetramerization on the reaction rate arises from alterations in steric and electrostatic properties of an active site due to the presence of an opposing subunit. However, the structural dynamics such as inter-subunit motions mitigate the effect of tetramerization and allow the enzyme to maintain a similar level of efficiency in different oligomerization states. As a result, the rate per active site calculated for some of the tetramer structures extracted from the MD trajectories is only approximately 15% smaller than the rate in the monomer. The conclusion from this study is that the near diffusion-controlled rate of hydrolysis of ACh catalyzed by *h*AChEt involves an intricate interplay of dynamic, electrostatic and steric effects, while the structural dynamics minimizes the adverse effect of tetramerization by modulating the electrostatic and steric effects, allowing the enzyme to maintain similar enzymatic efficiency in different oligomerization states. 

### 2.5. Catalytic Reaction Mechanism

AChE is one of the most efficient enzymes in nature. There is, therefore, considerable interest in characterization of the catalytic reaction mechanism and understanding the structural origin of the catalytic efficiency of the enzyme. Like most of serine hydrolases or proteases, the catalytic process of AChE includes two successive stages, acylation and deacylation. Zhang et al. first studied the initial step of the acylation reaction by combined ab initio QM/MM. QM/MM is a hybrid computational method that aims to combine the strengths of the QM (accuracy) and MM (speed) approaches, through exerting a QM calculation on the ligand binding interactions, the core region, and a MM calculation on the rest of the system [92,93]. Given that the QM/MM approach is able to explore chemical bond formation, it can be utilized to study the catalytic mechanism of an enzyme and provide useful information for the rational design of mechanism-based inhibitors. In the acylation process, the reaction proceeds through the nucleophilic addition of the hydroxyl oxygen of S203 in *m*AChE to the carbonyl carbon of ACh, simultaneously facilitated by proton transfer from S203 to H447, which serves as a general base. The calculated potential energy is in accordance with the experimental reaction rate. Through electrostatic interactions, the major roles of E334, the third residue of the catalytic triad, and of the oxyanion hole, formed by NH groups of G121, G122 and A204, are to stabilize the transition state and the tetrahedral intermediate, respectively. Several years later, Zhang et al. performed a second QM/MM study on characterization of the complete catalytic reaction mechanism and determination of the multistep free-energy reaction profiles of ACh hydrolysis by employing Born-Oppenheimer MD simulations with a B3LYP/6-31G(d) QM/MM potential and the umbrella sampling method [94]. In this study, the activation energy barriers for the acylation and deacylation reaction stages were calculated, with values of 12.4 kcal/mol and 17.5 kcal/mol, respectively, and it was indicated that the initial step of deacylation is the rate-limiting step. From the intermediate to the product, the orientation of the His447 ring needs to be adjusted slightly, and then proton transfer from His447 to the product and the breakage of the scissile bond happen spontaneously without any activation barriers. The three-pronged oxyanion hole only makes two hydrogen bonds with the carbonyl oxygen in the initial reactant and final product states, but the third hydrogen bond is formed and stable at all transition and intermediate states during the catalytic process.

Using the same approach, Zhang’s research group also revealed molecular mechanistic insights into the interaction of the nerve agent soman with AChE [95,96]. As mentioned above, the catalytic serine of AChE is rapidly phosphonylated by soman, which is the most potent nerve agent, and the conjugate subsequently undergoes an aging reaction, resulting in irreversible inhibition of AChE (Figure 3). The reaction appears to employ a highly coupled addition-elimination mechanism. It starts with the addition step in which H440 acts as a general base to facilitate the nucleophilic attack of the hydroxyl oxygen of S200 at soman’s phosphorus atom to form a trigonal bipyrimidal pentacovalent intermediate. Subsequently, cleavage of the phosphorus–fluorine covalent bond results in a stable soman–AChE covalent conjugate and a negatively charged fluorine atom that is stabilized by interactions with the hydroxyl group of Y121 before its dissociation from the active site. This is the so-called elimination step. Analogously, Bennion et al. reported a water assisted addition-elimination mechanism for soman-*h*AChE reaction based on QM/MM together with 80 classical MD simulations of the apo and soman-conjugated forms of *h*AChE [59]. Zhang et al. presented evidence for involvement of other residues in the aging process, besides those of the catalytic triad. For example, the process is favored by the protonated state of E199 in *Tc*AChE [95]. Besides E199, other non-catalytic residues including W84, Y130 and E443 also affect the dealkylation reaction of the soman-inhibited *Tc*AChE through direct interaction with the covalently bound soman or by modulating the side-chain orientation of other adjacent residues [97].

The reaction mechanism of two inhibitors TFK^+^ and TFK^0^, binding to both wild-type and H447I mutant *m*AChE have been investigated by using the ab initio QM/MM approach combined with classical MD simulations [98,99]. The reactions of TFK^+^ and TFK^0^ with the wild-type *m*AChE both proceed through the nucleophilic addition of the O_Υ_ of S203 to the carbonyl carbon of TFK^+^ or TFK^0^, accompanied by simultaneous proton transfer from S203 to H447, with no energy barrier found. This is consistent with the experimental reaction rates that approach the diffusion-controlled limit. By contrast, when TFK^+^ binds to the H447I mutant *m*AChE, a water molecule takes over the role of H447 and participates in the bond breaking. A second difference is that in the case of the mutant the catalytic triad residue E334 acts as the catalytic base in the reaction since H447 has been mutated. The calculated energy barrier for this reaction is about 8 kcal/mol. In the multiple MD simulations of the H447I mutant in complex with TFK^0^, none of the water molecules were retained in the active site as a “catalytic” water. The calculated binding free energy of TFK^0^ to H447I is much weaker than that of TFK^+^, and TFK^0^ is thus almost inactive towards the H447I mutant.

## 3. Concluding Remarks

In this review, we first surveyed computational simulation studies that reveal the dynamic features of the active-site gorge. The behavior of the key elements of the gorge including the gate (bottleneck), the strong electrostatic dipole along the gorge axis, multiple flexible aromatic residues, the buried water molecules, and the mobile Ω-loop, as well as their involvement in binding of ligands to the enzyme were discussed. Beside the active-site gorge as a main passageway for substrates, products, and other ligands, other trafficking pathways for ligands, such as back doors and side doors, were detected in numerous MD simulations. We then presented the detailed trafficking pathways and interactions for several ligands, including those for the substrates, products and their mimics, small molecule inhibitors, such as HupA and E2020, the polypeptide toxin inhibitor, FAS2, and oximes. Subsequently, the dynamic features of oligomeric AChEs are reviewed, since functional forms of this enzyme are often oligomers. Finally, we reviewed theoretical studies on the reaction mechanisms for hydrolysis of the natural substrate, ACh, and for irreversible inhibition by soman, followed by the subsequent “aging” reaction of the soman-AChE conjugate, which involved use of QM/MM calculations together with MD simulations. Taken together, these computational studies show how the dynamic nature of AChE is intimately linked to its function, and reveal, at an atomic level, the mechanisms underlying its function from a dynamic perspective. The emergent picture is that AChE appears to have been shaped by evolution in a number of ways to operate with unusual speed and robustness. The complexity of its structure/function relationships is far from being totally elucidated. This comprehensive review of computational studies on AChE serves as a paradigm for how multiple simulation methods, complemented by experimental studies, provide a powerful tool for understanding complex macromolecular systems.

## Figures and Tables

**Scheme 1:** The catalytic action of AChE on ACh.

(1) $${\mathrm{CH}}_{3}{\mathrm{COOCH}}_{2}{\mathrm{CH}}_{2}N^{+}{\left({\mathrm{CH}}_{3}\right)}_{3}+\mathrm{AChE}\to {\mathrm{HOCH}}_{2}{\mathrm{CH}}_{2}N^{+}{\left({\mathrm{CH}}_{3}\right)}_{3}+{\mathrm{CH}}_{3}\mathrm{CO}-\mathrm{AChE}$$

(2) $${\mathrm{CH}}_{3}\mathrm{CO}-\mathrm{AChE}+H_{2}O\to {\mathrm{CH}}_{3}{\mathrm{COO}}^{-}+H^{+}+\mathrm{AChE}$$
