# Erratum: Xu, Y., et al. Computational Studies on Acetylcholinesterases. *Molecules* 2017, *22*, 1324

**DOI:** 10.3390/molecules22091486

**Published:** 2017-09-06

**Authors:** Molecules Editorial Office

**Affiliations:** MDPI AG, St. Alban-Anlage 66, 4052 Basel, Switzerland; molecules@mdpi.com

The *Molecules* Editorial Office wishes to make the following erratum to this paper [1]. There is a typing error in the catalytic triad of *Tc*AChE within the text (line 9, page 2) as well as in the legend of Figure 1 (line 6 from the bottom, page 3). The correct catalytic triad is “H440-E327-S200”.

We apologize for any inconvenience caused to the readers by this mistake. The change does not affect the scientific results.
